# The latent period of coronavirus disease 2019 with SARS‐CoV‐2 B.1.617.2 Delta variant of concern in the postvaccination era

**DOI:** 10.1002/iid3.664

**Published:** 2022-06-10

**Authors:** Tao Ma, Songning Ding, Rui Huang, Hengxue Wang, Junjun Wang, Jiacheng Liu, Jian Wang, Jie Li, Chao Wu, Huafeng Fan, Nan Zhou

**Affiliations:** ^1^ Department of Acute Infectious Disease Control and Prevention Nanjing Municipal Center for Disease Control and Prevention Nanjing China; ^2^ Department of Infectious Diseases, Nanjing Drum Tower Hospital The Affiliated Hospital of Nanjing University Medical School Nanjing China; ^3^ Department of Infectious Diseases, Nanjing Drum Tower Hospital Clinical College of Traditional Chinese and Western Medicine Nanjing University of Chinese Medicine Nanjing Jiangsu China; ^4^ Microbiology Laboratory Nanjing Municipal Center for Disease Control and Prevention Nanjing China

**Keywords:** latent period, severe acute respiratory syndrome coronavirus 2, vaccine, variant of concern

## Abstract

**Introduction:**

Emerging variants of severe acute respiratory syndrome coronavirus 2 (SARS‐CoV‐2) have resulted in new challenges for epidemic prevention and control worldwide. However, little is known about the latent period of coronavirus disease by the SARS‐CoV‐2 Delta variant of concern (VOC) in the postvaccination era.

**Methods:**

The epidemiology and clinical data of cases with confirmed SARS‐CoV‐2 Delta VOC infection were retrospective collected. Dates of the first positive PCR test were collected to estimate the distribution of latent period.

**Results:**

Of the 40 patients, 16 were male (40%). The median age of patients was 47.5 years. The median latent period of patients was 6.0 days (interquartile range [IQR], 4.0−9.0 days) and the longest latent period was 13.0 days after exposure. The latent periods were longer in male patients compared to female patients (median, 8.5 days vs. 5.0 days, *p* = .041). The median latent period was comparable among fully vaccinated cases (6.5 days), no vaccinated cases (7.5 days), and partially vaccinated cases (5.5 days).

**Conclusions:**

The median latent period of SARS‐CoV‐2 Delta VOC infection was 6.0 days. The latent period between vaccinated and non‐vaccinated patients was not significantly different. The 14‐day quarantine program is sufficient to prevent the transmission of COVID‐19 by Delta VOC in the postvaccination era.

## Graphical abstract

The median latent period of SARS‐CoV‐2 Delta variant of concern (VOC) infection was 6.0 days. No significant difference of the latent period between vaccinated and non‐vaccinated patients was found. Our study support that the 14‐day quarantine program is sufficient to prevent the transmission of COVID‐19 by Delta VOC in the postvaccination era.

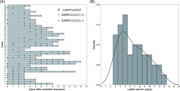

## 1 INTRODUCTION

Emerging variants of severe acute respiratory syndrome coronavirus 2 (SARS‐CoV‐2) have resulted in new challenges for epidemic prevention and control worldwide. The SARS‐CoV‐2 lineage B.1.617.2 (Delta) variant of concern (VOC) was a variant with faster spread compared to other variants.^1^

A number of studies have estimated the incubation period of wild‐type.^2^, ^3^ However, contrasted with the incubation period, the latent period is the time interval between infection and onset of infectiousness which is important for the control of infectious disease transmission.^4^ Xin et al.^5^ had estimated the latent period of SARS‐CoV‐2 infection, while little is known about the latent period of coronavirus disease by the SARS‐CoV‐2 Delta VOC in the postvaccination era.

## 2 METHODS

The time of exposure to SARS‐CoV‐2 Delta VOC, any symptom onset, SARS‐CoV‐2 vaccination information, and case detection of each patient with confirmed SARS‐CoV‐2 Delta VOC infection were retrospectively collected. Data on the exposure to infection were collected to estimation of the latent period distribution. Repeated testing results and sampling dates of each PCR test were collected. Dates of the first positive PCR test were collected to estimate the latent period distribution. Vaccination status of patients with COVID‐19 was categorized into three groups according to the immunization history of SARS‐CoV‐2 vaccine, which included fully vaccinated (two doses of SARS‐CoV‐2 vaccines), partially vaccinated (single dose of SARS‐CoV‐2 vaccine) and no vaccinated (no SARS‐CoV‐2 vaccine doses). This study was approved by the Institutional Review Board at Nanjing Municipal Center for Disease Control and Prevention with a waiver of informed consent.

## 3 RESULTS

**Figure 1:**
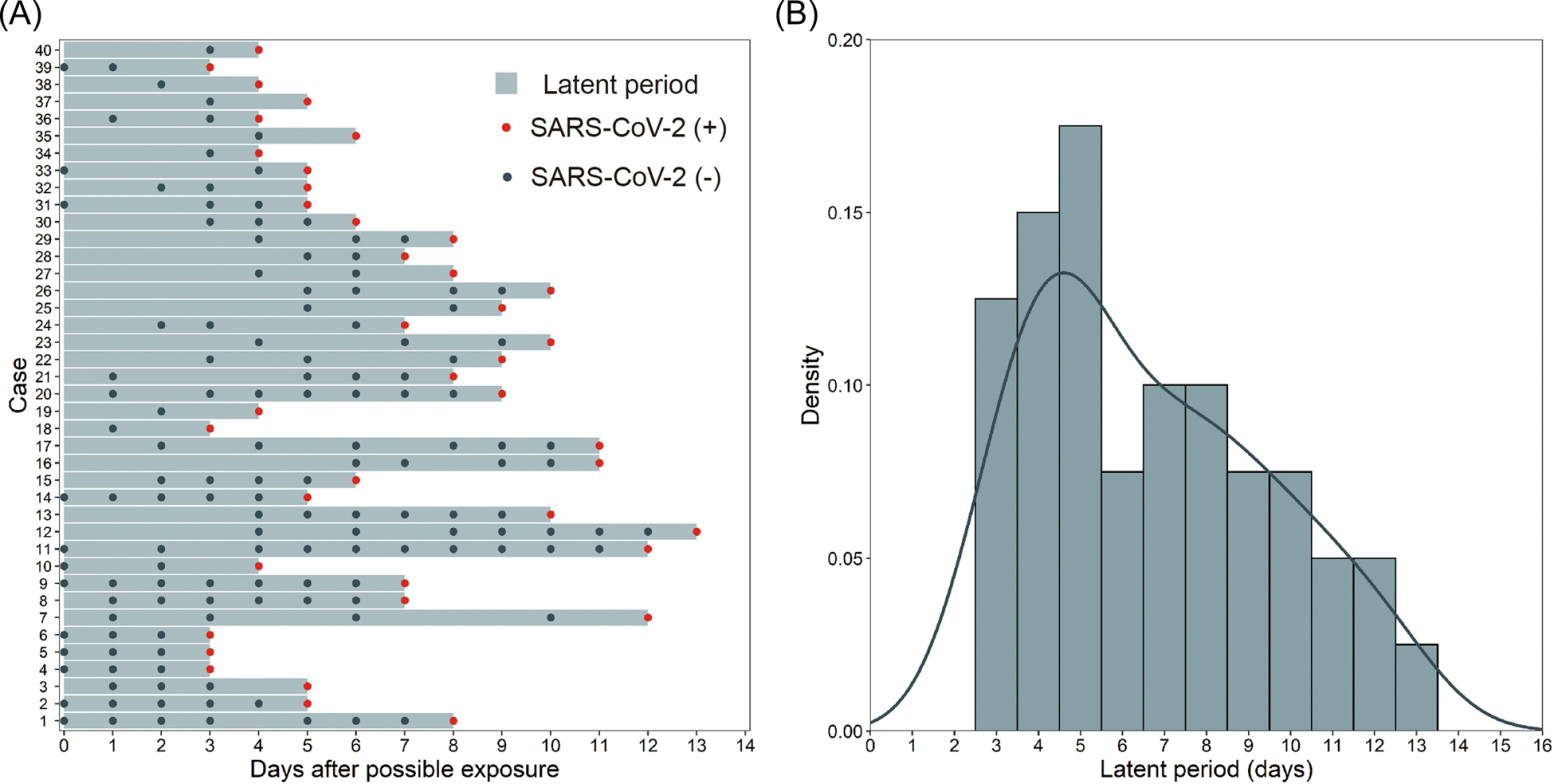
Latent period of coronavirus disease 2019 with SARS‐CoV‐2 B.1.617.2 Delta variant of concern. SARS‐CoV‐2 Delta VOC exposure and case detection times (A) and latent period distribution (B).

Forty cases with confirmed SARS‐CoV‐2 Delta VOC infection were collected (Table 1). Of these, 16 were male (40%). The median age was 47.5 years (interquartile range (IQR), 32.3–55.0 years). All cases had a known history of single confirmed exposure. The timelines of disease onset in confirmed COVID‐19 patients were presented in Figure 1A. The median times of SARS‐CoV‐2 RNA screening before the first positive test result was three times. The median latent period was 6.0 days (IQR, 4.0–9.0 days) after exposure. The longest latent period was 13.0 days (Figure 1B) after exposure.

**Table 1:** Characteristics of patients with SARS‐CoV‐2 Delta VOC infection

Characteristics	Median (IQR) or *n* (%)
**Age**	
≤18	6 (15)
19–60	26 (65)
≥61	8 (20)
**Gender**	
Male	16 (40)
Female	24 (60)
**Comorbidities**	
Type 2 diabetes	3 (7.5)
Cardiovascular diseases	9 (22.5)
Chronic liver diseases	1 (2.5)
**Onset symptoms**	
Fever	11 (27.5)
Cough	21 (52.5)
Fatigue	4 (10.0)
Sore throat	6 (15.0)
Diarrhea	2 (5.0)
Headache	1 (2.5)
Vomit	2 (5.0)
Muscle ache	4 (10.0)
**Severity of illness**	
Non‐severe illness	37 (92.5)
Severe illness	3 (7.5)
**Vaccination status**	
No vaccinated	12 (30)
Partially vaccinated	14 (35)
Fully vaccinated	14 (35)

Abbreviations: IQR, interquartile range; VOC, variant of concern.

**Figure 2:**
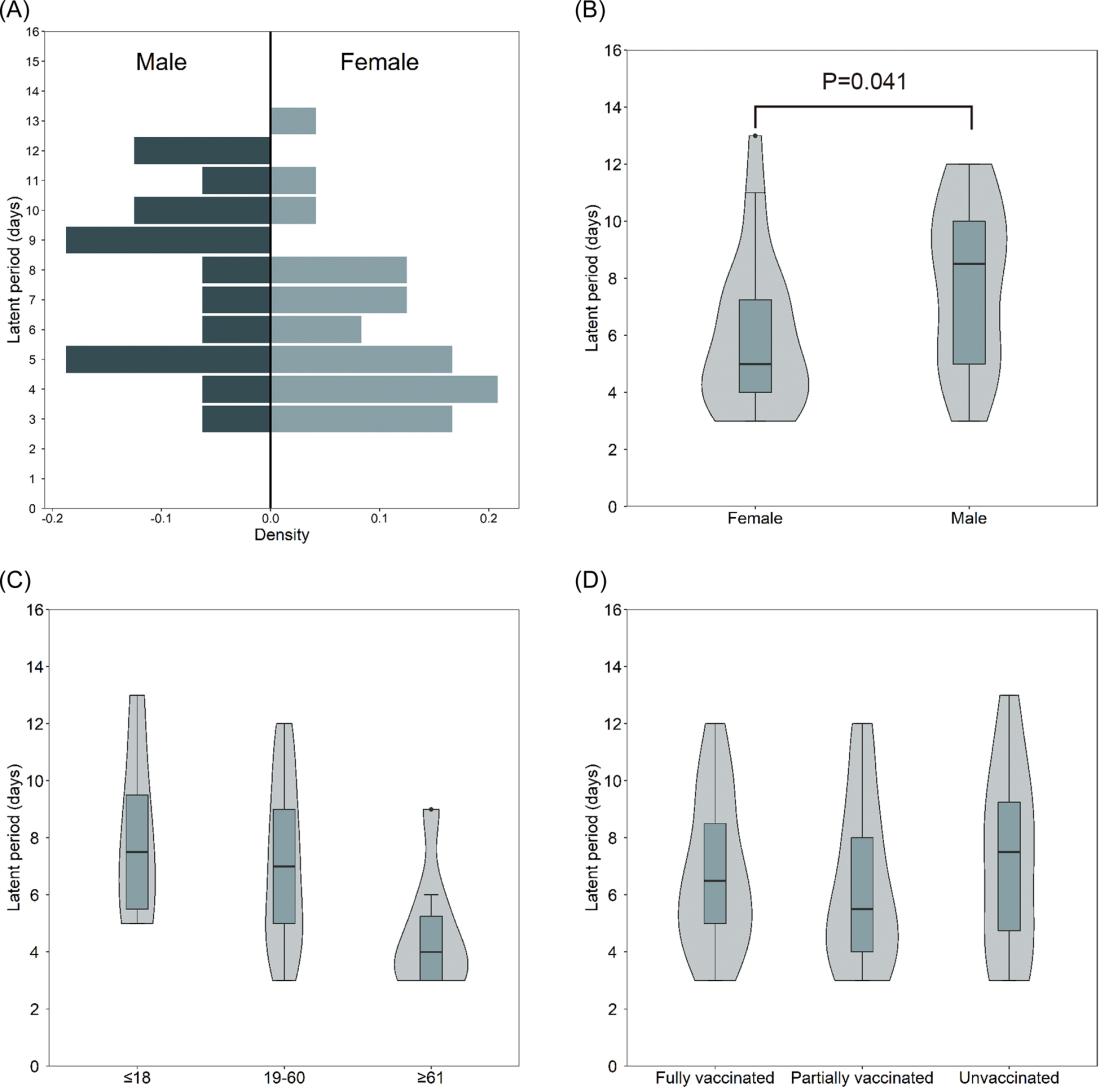
Comparisons of the latent period distribution in COVID‐19 patients with different gender (A,B), age (C), and vaccination status (D)

The latent periods were longer in male patients compared to female patients (median, 8.5 vs. 5.0 days, *p* = .041; Figure 2A,B). The latent periods were comparable between different age groups (Figure 2C). Fourteen patients had received full vaccination. The median latent period among fully vaccinated cases was 6.5 days (IQR, 4.8–9.3 days) which was similar to the no vaccinated cases and partially vaccinated cases with the median latent periods of 7.5 days (IQR, 4.3–9.8 days, *p* = .697) or 5.5 days (IQR, 4.0–8.3 days, *p* = .701), respectively (Figure 2D).

## 4 DISCUSSION

Comprehensive large‐scale SARS‐CoV‐2 RNA testing strategies have been widely used in mainland China to control outbreaks of COVID‐19.^6^ Close contacts of confirmed cases were required to be quarantined for 14 days and samples were repeatedly collected to screen for SARS‐CoV‐2 RNA regardless of whether any symptoms were reported, providing a possibility for the calculation of latent period.^5^ Xin et al.^5^ estimated the mean latent period of COVID‐19 to be 5.5 days (95% confidence interval: 5.1–5.9 days). However, the presence of variants of SARS‐CoV‐2 in the study was not described.

To the best of our knowledge, our study is the first study that estimated the latent period of COVID‐19 with Delta VOC of 6 days. Only patients with single exposure were included. Moreover, patients were repeated tested every day or every other day for SARS‐CoV‐2 RNA before confirmed COVID‐19. Thus, we considered that the latent period is accurate. We found that the latent period was shorter in female patients compared to male patients. However, the latent period was comparable between vaccinated patients and no vaccinated patients.

This study presents some limitations. First, the sample size used to estimate the latent period was small since we only included cases with single possible exposure. Patients with multiple exposures, continued exposure, or no known exposure were excluded from the analysis. Second, we did not consider the demographic characteristics of the patients, which may have affected the latent period.

In conclusion, the median latent period of SARS‐CoV‐2 Delta VOC infection was 6.0 days. No significant difference in the latent period between vaccinated and non‐vaccinated patients was found. Our study support that the 14‐day quarantine program is sufficient to prevent the transmission of COVID‐19 by Delta VOC in the postvaccination era.

## AUTHOR CONTRIBUTIONS

All authors contributed to this study at different levels. *Study concept and design*: Nan Zhou, Huafeng Fan, and Rui Huang. *Acquisition of data*: Hengxue Wang, Junjun Wang, Jiacheng Liu, Jian Wang, Jie Li, and Chao Wu. *Statistical analysis and interpretation of data*: Rui Huang and Hengxue Wang. *Drafting of the manuscript*: Tao Ma, Songning Ding, and Rui Huang. *Critical revision of the manuscript for important intellectual content*: Nan Zhou, Huafeng Fan, and Rui Huang.

## References

[1] WHO . Weekly Epidemiological Update on COVID‐19 – 11 May 2021. May 11, 2021. Accessed November 8, 2021. https://www.who.int/publications/m/item/weekly-epidemiological-update-on-covid-19---11-may-2021

[2] Lauer SA , Grantz KH , Bi Q , et al. The incubation period of coronavirus disease 2019 (COVID‐19) from publicly reported confirmed cases: estimation and application. Ann Intern Med. 2020;172(9):577‐582. 32150748 10.7326/M20-0504 PMC7081172

[3] McAloon C , Collins Á , Hunt K , et al. Incubation period of COVID‐19: a rapid systematic review and meta‐analysis of observational research. BMJ Open. 2020;10(8):e039652. 10.1136/bmjopen-2020-039652 PMC7430485 32801208

[4] Fine PE . The interval between successive cases of an infectious disease. Am J Epidemiol. 2003;158(11):1039‐1047. 14630599 10.1093/aje/kwg251

[5] Xin H , Li Y , Wu P , et al. Estimating the latent period of coronavirus disease 2019 (COVID‐19). Clin Infect Dis. 2022;74(9):1678‐1681. 34453527 10.1093/cid/ciab746

[6] Li Z , Liu F , Cui J , et al. Comprehensive large‐scale nucleic acid‐testing strategies support China's sustained containment of COVID‐19. Nat Med. 2021;27(5):740‐742. 33859409 10.1038/s41591-021-01308-7

